# Inhibition of colorectal cancer stem cell survival and invasive potential by hsa-miR-140-5p mediated suppression of Smad2 and autophagy

**DOI:** 10.18632/oncotarget.3771

**Published:** 2015-04-27

**Authors:** Haiyan Zhai, Andrew Fesler, Yufeng Ba, Song Wu, Jingfang Ju

**Affiliations:** ^1^ Translational Research Laboratory, Department of Pathology, Stony Brook University, Stony Brook, NY 11794 USA; ^2^ Department of Thoracic Surgery, The Cancer Hospital of Henan, Zhengzhou, Henan 450008 China; ^3^ Department of Applied Mathematics and Statistics, Stony Brook University, Stony Brook, NY 11794 USA

**Keywords:** hsa-miR-140-5p, smad2, autophagy, metastasis, colon cancer stem cell

## Abstract

Colorectal cancer (CRC) is the third highest mortality cancer in the United States and frequently metastasizes to liver and lung. Smad2 is a key element downstream of the TGF-β signaling pathway to regulate cancer metastasis by promoting epithelial to mesenchymal transition and maintaining the cancer stem cell (CSC) phenotype. In this study, we show that hsa-miR-140-5p directly targets Smad2 and overexpression of hsa-miR-140-5p in CRC cell lines decreases Smad2 expression levels, leading decreased cell invasion and proliferation, and increasing cell cycle arrest. Ectopic expression of hsa-miR-140-5p in colorectal CSCs inhibited CSC growth and sphere formation *in vitro* by disrupting autophagy. We have systematically identified targets of hsa-miR-140-5p involved in autophagy. Furthermore, overexpression of hsa-miR-140-5p in CSCs abolished tumor formation and metastasis *in vivo*. In addition, there is a progressive loss of hsa-miR-140-5p expression from normal colorectal mucosa to primary tumor tissues, with further reduction in liver metastatic tissues. Higher hsa-miR-140 expression is significantly correlated with better survival in stage III and IV colorectal cancer patients. The functional and clinical significance of hsa-miR-140-5p suggests that it is a key regulator in CRC progression and metastasis, and may have potential as a novel therapeutic molecule to treat CRC.

## INTRODUCTION

Colorectal cancer (CRC) is one of the most common malignancies and causes 655,000 deaths per year worldwide [1]. The most frequent sites of metastasis for CRC are liver and lung. Many early detection methods and therapeutic approaches have been explored, which have led to steady improvements in treating metastatic CRC over the last 20 years. However, the 5-year survival rate is still around 10% for the stage IV advanced stage colorectal cancer [2]. One of the major reasons for the failure of treating metastatic colorectal cancer is chemoresistance, and colorectal cancer stem cells (CSCs) are the key components of the resistance mechanism.

In addition to resistance, increasing evidence suggests that tumor initiation and metastases are also dependent on the small sub-population of CSCs [3]. Human colorectal CSCs were first isolated by sorting CRC cells based on CD133 expression, and these high CD133-expressing cells were demonstrated to induce tumors in mice resembling the original malignancy [4, 5]. Further analysis has shown that CSCs have an increased ability of self-renewal, chemoresistance, and seeding secondary tumors [4–7]. Many cellular signaling pathways have been studied to explore the mechanism of invasion and metastasis of CSC, and the transforming growth factor β (TGF-β) signaling pathway has been shown to be one of the most important regulatory networks.

Smad2 is one of the key components downstream of the TGF-β signaling pathway. TGF-β exerts its effects by acting on two types of transmembrane receptors: type I (TGFBRI) and type II (TGFBRII). Activation of the TGFBRI invokes several TGF-β signaling pathway targets and downstream genes, including Smad2 and Smad3, which translocate to the nucleus and act as transcription factors [8, 9]. Smad2 is overexpressed in CRC cancer tissue as compared to normal colorectal mucosa [10, 11], and plays an important role in promoting tumor progression. It serves as a direct mediator of TGF-β signaling regulating multiple aspects of cancer progression, including immune suppression, angiogenesis, apoptosis, cell growth, and epithelial to mesenchymal transitions (EMT) [12]. In advanced CRC, increased TGF-β levels are associated with poor prognosis due to the induction of Smad2 accumulation in tumor stromal cells, which results in increased survival of metastatic cells and organ colonization [13]. Moreover, Smad2 and Smad3 can be activated by Nodal to promote colorectal CSCs self-renewal and CRC carcinogenesis [14]. By increasing CSC features, cancer cells would have an advantage to survive in nutrient starvation conditions and colonize distant organs. It has been shown that inhibitors that decrease levels of Smad2 and Smad3 through interruption of TGF-β signaling could be valuable tools for treatment of human cancers such as CRC, glioblastoma and breast cancer [15–19].

Limited studies have been reported on the involvement of non-coding microRNAs (miRNAs) in Smad2 signaling in colorectal cancer stem cells. miRNAs are non-coding RNA molecules, 18-25 nucleotides in length, that regulate the expression of their target genes by translational arrest or mRNA cleavage, most likely, through binding to 3′-UTRs of the target mRNAs [20, 21]. miRNAs have been found to regulate many cellular processes including apoptosis [22–25], differentiation [21, 26, 27], and cell proliferation [22, 27–29]. Hsa-miR-140-5p has been shown to inhibit TGFBRI in hepatocellular carcinoma (HCC), and its overexpression can suppress HCC growth and metastasis [30]. Down-regulation of hsa-miR-140-5p could promote CSC formation in breast cancer [31], and promote invasion in esophageal cancer [32]. We have shown that ectopic expression of hsa-miR-140-5p in CRC cells can inhibit cell growth and induce cell cycle arrest, in part, by suppressing the expression of HDAC4 [33]. However, the comprehensive mechanism and impact of hsa-miR-140-5p on CRC invasion, metastasis, and colorectal CSC survival still remains elusive.

In this study, we discovered novel mechanisms and targets of hsa-miR-140-5p in colorectal CSCs. We show that hsa-miR-140-5p directly suppressed Smad2 expression, and overexpression of hsa-miR-140-5p inhibited cell invasion, proliferation, and induced cell cycle arrest. Ectopic expression of hsa-miR-140-5p in colorectal CSCs inhibited CSC growth and sphere formation *in vitro* by disrupting autophagy, and abolished the tumor formation and metastasis *in vivo* in NOD/SCID mice. Moreover, we found a progressive loss of hsa-miR-140-5p expression from normal colorectal mucosa to primary tumor tissues, with further reduction in liver metastatic tissues, suggesting that hsa-miR-140-5p is a key regulator in maintaining colorectal cancer stemness phenotype and colorectal cancer progression and metastasis.

## RESULTS

### Smad2 is a direct target of hsa-miR-140-5p in colon cancer cells

**Figure 1:**
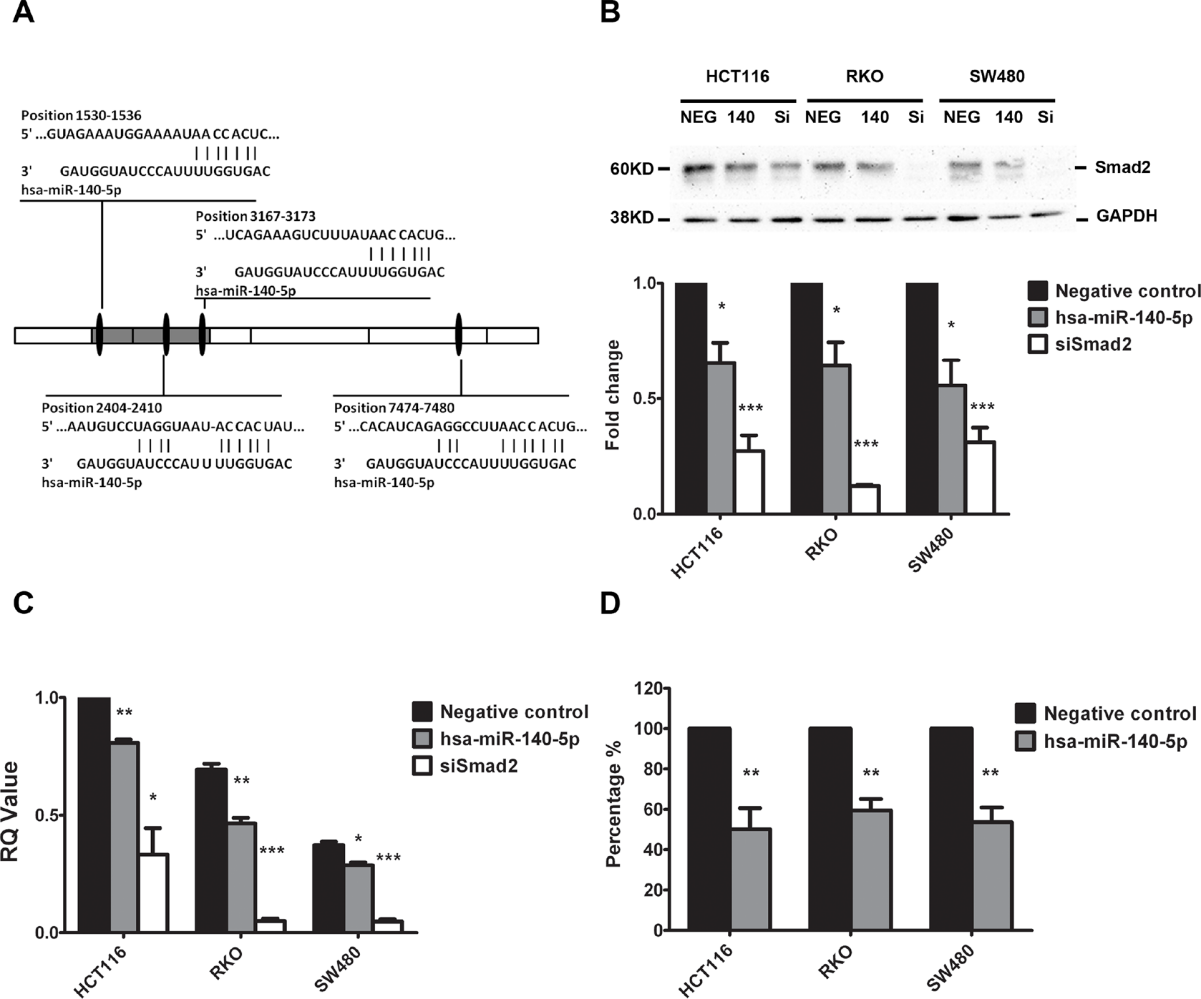
Smad2 is the direct target of hsa-miR-140-5p Four putative hsa-miR-140-5p binding sites exist in the 3′-UTR of Smad2 mRNA **A.** Ectopic expression of hsa-miR-140-5p (140) or siRNAs against Smad2 (siSmad2, Si) in HCT116, RKO and SW480 cells decreased Smad2 protein levels by Western blot analysis **B.** and mRNA level by real-time qRT-PCR **C.** Transfection of hsa-miR-140-5p inhibited firefly luciferase activity of pMIR-REPORT-3UTRSmad2 **D.** The negative miRNA (NEG) was used as the negative control in all experiments. The impact of hsa-miR-140-5p and siSmad2 on Smad2 expression was normalized and compared to those of negative miRNA (*n* = 3).

It has been shown that Smad2 contributes to cancer initiation, invasion, metastasis and CSC self-renewal [12, 14]. We have identified 4 putative hsa-miR-140-5p binding sites in the 3′-UTR of Smad2 mRNA using TargetScan analysis (Figure 1A). To experimentally confirm that Smad2 is a direct target of hsa-miR-140-5p, we transfected colon cancer cell lines, HCT116, RKO and SW480, with either negative control miRNA or hsa-miR-140-5p precursors, and quantified the expression of Smad2 at both protein and mRNA levels by western blot analysis and real-time qRT-PCR analysis, respectively. Our results revealed that hsa-miR-140-5p specifically suppressed the protein expression of Smad2 in all the cell lines (Figure 1B) and reduced the Smad2 mRNA transcript level (Figure 1C) compared to the controls. Interestingly, we did not find any reduction in TGFBRI level at both mRNA and protein level (data not shown) as reported previously in HCC [30].

To further confirm the direct binding of hsa-miR-140-5p to the 3′-UTR of Smad2 mRNA, we cloned a 2 Kb fragment of the Smad2 3′-UTR containing 3 predicted hsa-miR-140-5p binding sites and inserted it downstream of a firefly luciferase gene in a luciferase reporter vector, which was then transfected into all three colon cancer cell lines along with hsa-miR-140-5p or negative control miRNA. Hsa-miR-140-5p significantly inhibited the luciferase activity compared to the negative control miRNA (Figure 1D), suggesting that hsa-miR-140-5p was able to bind directly to the 3′-UTR of Smad2 mRNA to suppress translation. Based on these results, we conclude that hsa-miR-140-5p directly regulates Smad2 expression in colon cancer cell lines.

In addition to Smad2, we systematically identified hsa-miR-140-5p mediated targets via microarray expression analysis by comparing mRNA profiles between the control and hsa-miR-140-5p transfected HCT116 colon cancer cells. We have listed 40 candidate mRNA targets cross validated using TargetScan analysis (Supplementary Table 2). A number of these genes are involved in autophagy.

### Hsa-miR-140-5p inhibits CRC cell invasion *in vitro*

**Figure 2:**
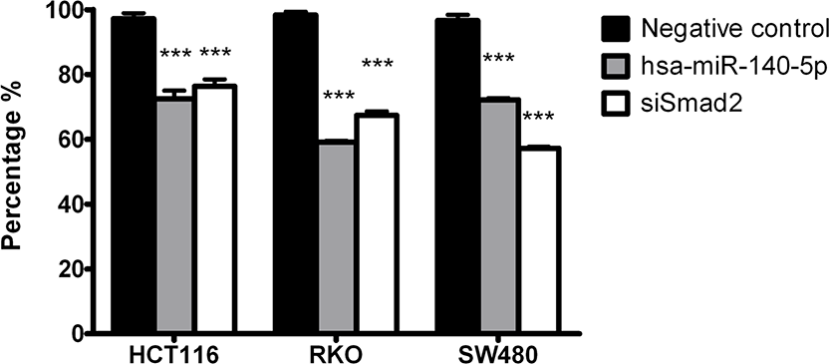
Hsa-miR-140-5p inhibits CRC cells invasion *in vitro* through Smad2 Ectopic expression of hsa-miR-140-5p in HCT116, RKO and SW480 cells decreased invasive activity measured by an ECM invasion assay. Knockdown of Smad2 by siRNAs has similar effects. (*n* = 3, *p* < 0.001).

Smad2 regulates EMT and promotes cancer cell invasion [8, 9], therefore we reasoned that hsa-miR-140-5p may have an impact on CRC invasion. We used a transwell system containing an extracellular matrix (ECM) layer to study the change in invasion activity. In all the CRC cell lines tested, we demonstrated that overexpression of hsa-miR-140-5p reduced cell number invading through the ECM to around 60% to 70% when compared to negative control (Figure 2). We further used siRNA against Smad2 to knockdown Smad2 expression (Figure 1B and 1C) and repeated the invasion assay, and we found that siSmad2 can also reduce the invasion activity to a similar level as hsa-miR-140-5p (Figure 2), suggesting that hsa-miR-140-5p impacts CRC invasion through Smad2.

### Hsa-miR-140-5p regulates CRC cell proliferation and cell cycle

**Figure 3:**
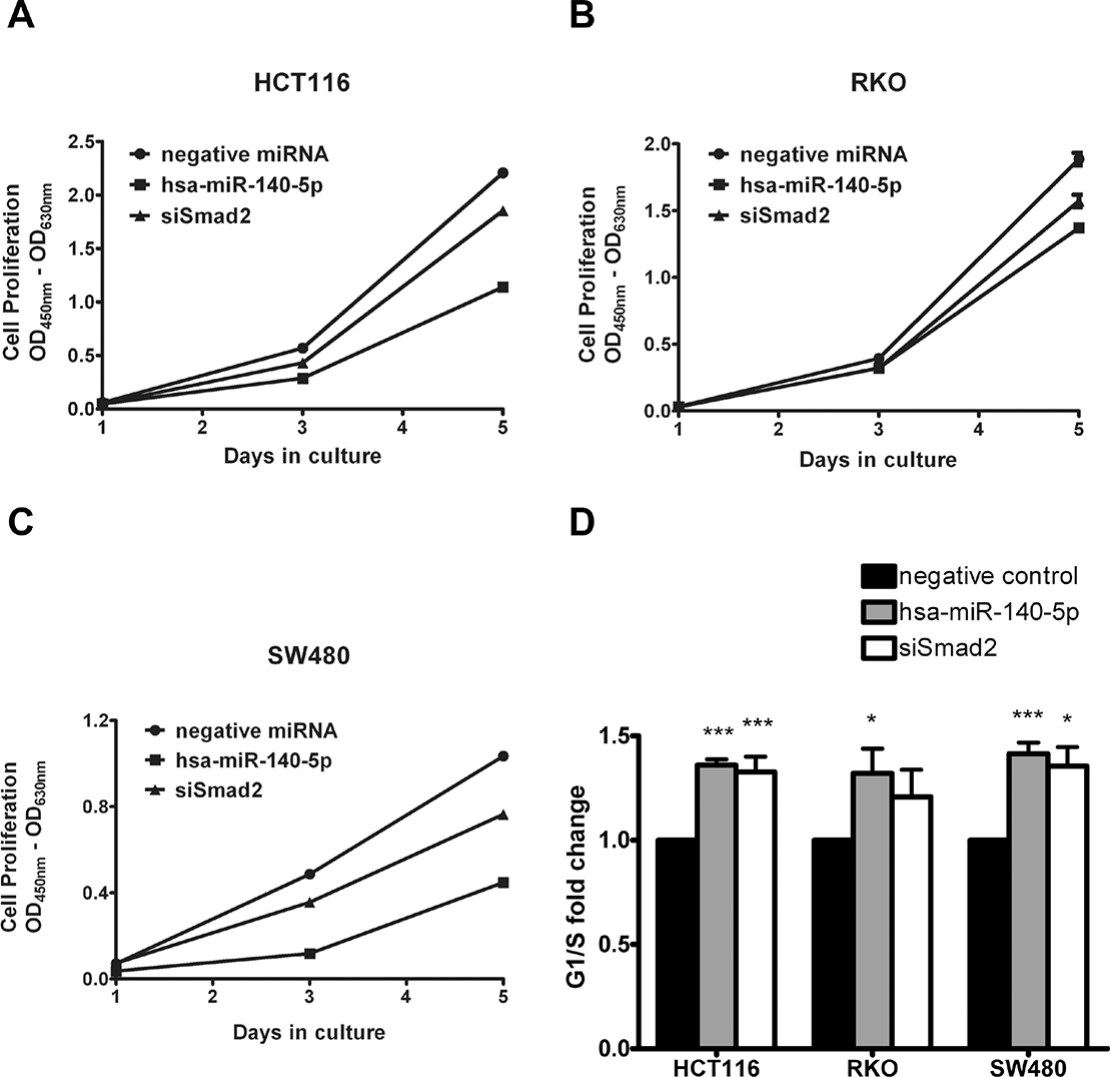
Hsa-miR-140-5p inhibited colon cancer cell growth and induced cell cycle arrest partially through targeting Smad2 HCT116 **A.** RKO **B.** and SW480 cells **C.** were transfected with hsa-miR-140-5p or siSmad2, and cell numbers were measured with WST-1 assay at day 1, 3 and 5. Cell cycle analysis was performed to determine the impacts of hsa-miR-140-5p and siSmad2. The G1/S ratio was shown in **D.** (*n* = 3).

Our group has reported that hsa-miR-140-5p can inhibit CRC cell proliferation and induce cell cycle arrest in HCT116 cells partially through the suppression of direct target HDAC4 [33]. It is well known that miRNAs exert their regulatory function through interactions with multiple direct targets; therefore we reasoned that Smad2 may also play a role in cell proliferation and cell cycle. We demonstrated that ectopic expression of hsa-miR-140-5p can significantly inhibit CRC cell growth, as the cell numbers of HCT116, RKO and SW480 overexpressing hsa-miR-140-5p were around 50% of the negative control group 5 days after transfection (Figure 3A). However, the growth inhibitory affect of hsa-miR-140-5p was contributed in part, by the suppression of Smad2 as siSmad2 can only account for a portion of the growth inhibitory effect (Figure 3A).

We further analyzed cell cycle control by flow cytometry and found that hsa-miR-140-5p overexpression led to an increase in G1 phase with a decrease in S phase (Supplementary Figure 2). The G1/S ratio (Figure 3B) indicated that hsa-miR-140-5p induced cell cycle arrest at G1 checkpoint. We demonstrated that the suppression of Smad2 contributed to cell cycle arrest of CRC cells by a siRNA knockdown based approach (Figure 3B).

### Hsa-miR-140-5p disrupts CSC growth through interrupting autophagy

It has been reported that hsa-miR-140 is downregulated in breast CSCs when compared to normal breast stem cells [31], and Nodal promotes colorectal CSC renewal through activation of Smad2 and Smad3 [14]. We reasoned that hsa-miR-140 may have an impact on colorectal CSC properties. We isolated colorectal CSCs by culturing HCT116 cells in serum-free media in ultra-low attachment tissue culture flasks for two weeks. The spheres collected from this process were confirmed to be CD44^high^CD133^high^ (Supplementary Figure 1A), and could form subcutaneous tumors in NOD/SCID mice at much lower cell number compared to parental HCT116 cells (Supplementary Figure 1B).

**Figure 4:**
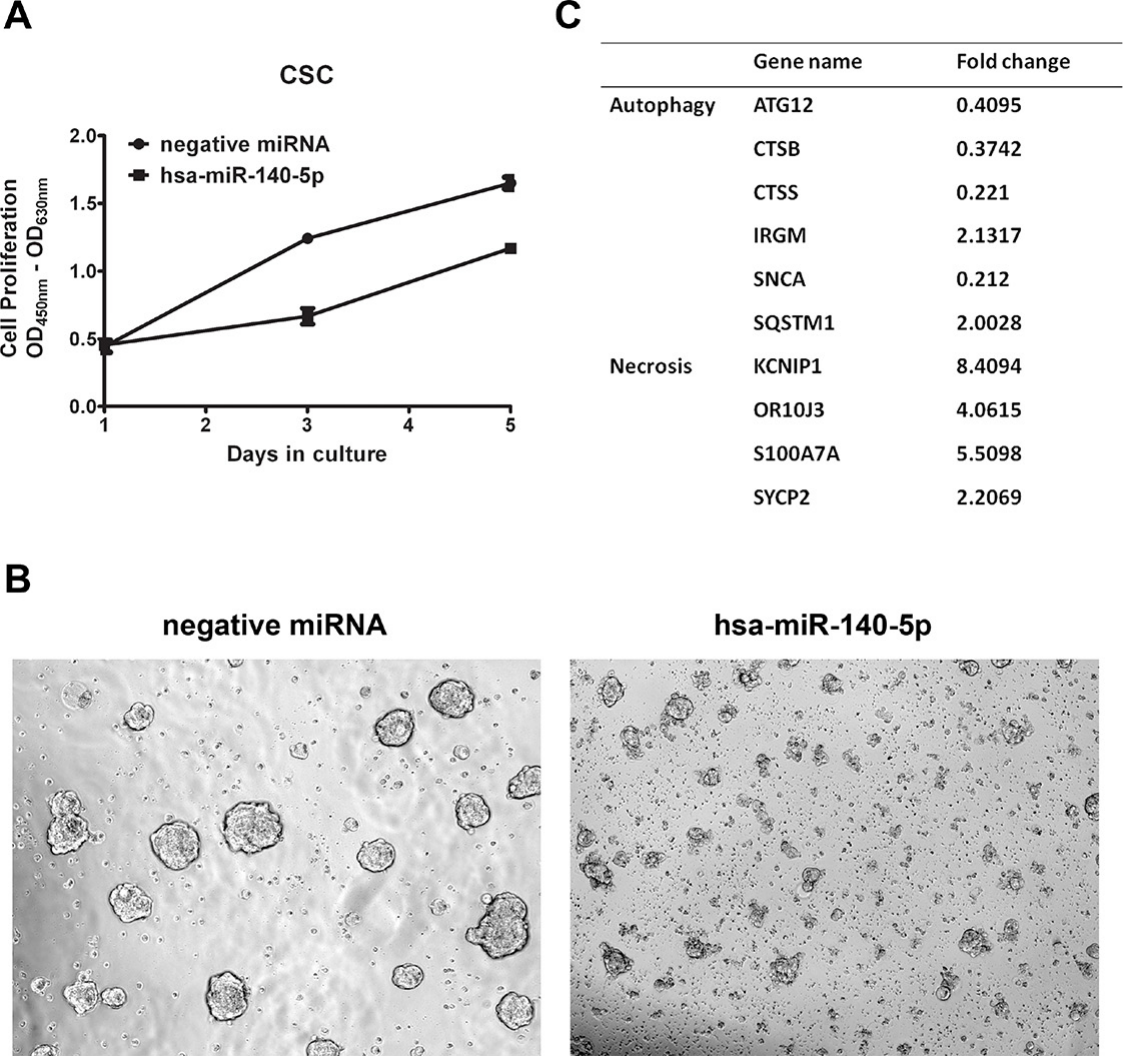
Hsa-miR-140-5p disrupts CSC growth through interrupting autophagy **A.** HCT116 CSCs were transfected with hsa-miR-140-5p, and cell numbers were measured with WST-1 assay at day 1, 3 and 5 (*n* = 3). **B.** Representative bight field pictures of morphological changes were taken at 3 days after transfection (20×). **C.** The cell-death related genes were screened with PCRarray and gene changes over 2 folds are listed.

We first performed transient transfection of hsa-miR-140-5p precursors in these CSCs and monitored changes in cell number for 5 days. We found that overexpression of hsa-miR-140-5p slowed down CSC growth (Figure 4A), and disrupted sphere formation (Figure 4B). We further looked into the mechanism of this inhibitory effect, but surprisingly there was no significant change in CSC markers, such as Snail, Twist, OCT4 and NANOG, quantified by qRT-PCR (Supplementary Figure 3).

It has been reported that TGF-β induces autophagy in cancer cells through Smad2/Smad3 signaling pathway [34–36], and autophagy is an essential metabolizing process for CSC origin and maintenance [37], we reasoned that hsa-miR-140-5p may impact the colorectal CSC survival. We used Cell Death PathwayFinder PCR Array to systematically screen the impacts of hsa-miR-140-5p on CSC cell death, including apoptosis, necrosis and autophagy. The screening results revealed that ectopic expression of hsa-miR-140-5p had minimal effect on apoptosis (data not shown). However, it induced significant changes in genes related to autophagy and necrosis (Figure 4C). In addition to Smad2, we found another important direct target of hsa-miR-140-5p involved in autophagy. ATG12 is an essential gene regulating autophagosome formation [38], and was found to be downregulated to 40% by hsa-miR-140-5p (Figure 4C). Consistently similar results were found in the microarray analysis of hsa-miR-140-5p overexpression in HCT116 cells (Supplementary Table 2). We further analyzed the 3′ UTR of ATG12 and found there is a predicted binding site for hsa-miR-140-5p at position 2945-2951. Therefore overexpression of hsa-miR-140-5p disrupted autophagy in CSC through both Smad2 and ATG12, and then impacts the downstream regulators of autophagy, such as cathepsin B (CTSB), cathepsin S (CTSS), immunity-related GTPase family M (IRGM), α-synuclein (SNCA) and sequestosome 1 (SQSTM1) (Figure 4C). Moreover, this interruption of autophagy further led to direct killing of CSCs as evidenced by the upregulation of necrosis markers (Figure 4C).

### Hsa-miR-140-5p impacts CRC initiation and metastasis *in vivo*

**Figure 5:**
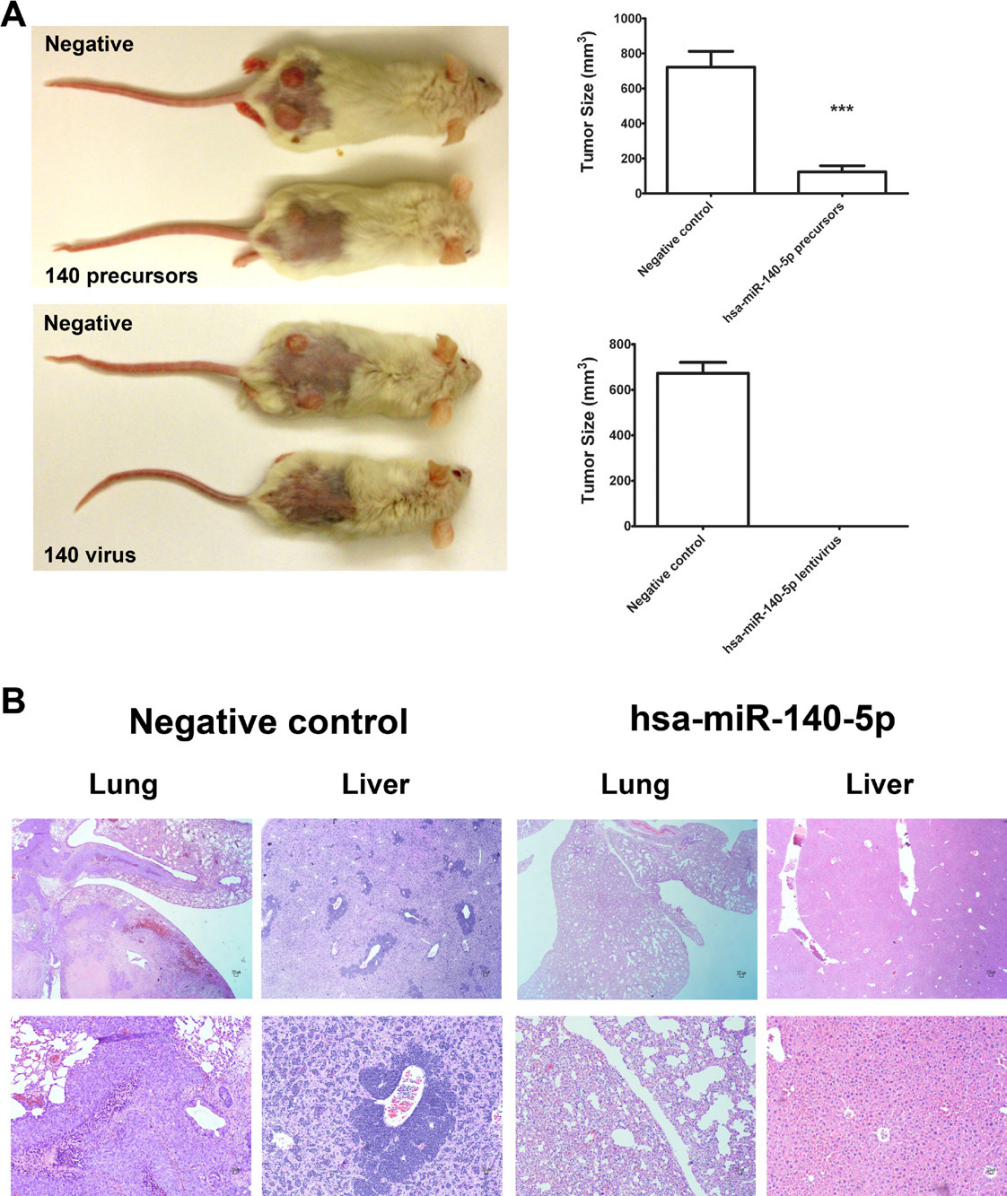
Hsa-miR-140-5p impacts CRC initiation and metastasis *in vivo* Prior to injection, HCT116 CSCs were either transfected with hsa-miR-140-5p precursors or infected with lentivirus expressing hsa-miR-140-5p. Corresponding negative controls were used for both treatments. **A.** 10^5^ HCT116 CSCs were subcutaneously injected into NOD/SCID mice. The tumor sizes were measured for 8 weeks and the final volumes are shown (*n* = 5). Representative images of mice bearing HCT116 tumors at 8 weeks after injection are shown. **B.** 10^5^ HCT116 CSCs were injected into NOD/SCID mice through tail vein. Lungs and livers were collected at 8 weeks after injection, and were subjected to H&E staining (*n* = 5). Representative images are shown (upper panel 10×, lower panel 20×).

Based on these *in vitro* inhibitory effects of hsa-miR-140-5p on CSCs, we further investigated the impacts in mouse CRC models. First, we explored the effects on tumor initiation by injecting CSCs subcutaneously on the back of NOD/SCID mice. Two days before injection, CSCs were either transfected with miRNA precursors or infected with lentiviral vectors expressing hsa-miR-140-5p. Then the subcutaneous tumor growth was monitored for 8 weeks. The CSCs transiently transfected with hsa-miR-140-5p precursors produced much smaller tumors than the negative control group (123.1 mm^3^ vs. 722.2 mm^3^) (Figure 5A), and the group stably overexpressing hsa-miR-140-5p failed to form tumors (Figure 5B).

Furthermore, we investigated the impacts of hsa-miR-140-5p on CSC metastasis by injecting CSCs into NOD/SCID mice through tail vein. Similarly CSCs were pre-treated as in the subcutaneous tumor formation experiment. 8 weeks later, the mice were euthanized, and the lung and liver were dissected out for H&E staining. We demonstrated that the negative control CSCs can form metastatic cancer colonies in both liver and lung, however, both groups overexpressing hsa-miR-140-5p by miRNA precursors or lentivirus (data not shown) failed to form lung or liver metastasis (Figure 5B). Therefore, hsa-miR-140-5p has a major impact on CRC initiation, invasion and metastasis.

### The expression levels of hsa-miR-140-5p are significantly correlated with CRC progression and survival

**Figure 6:**
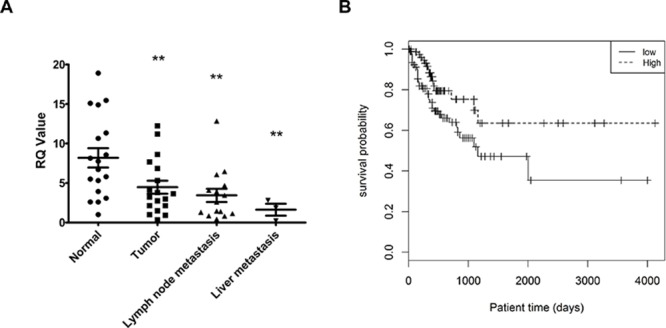
The expression level of hsa-miR-140-5p is significantly correlated with CRC patients survival **A.** Hsa-miR-140-5p was progressively downregulated in primary and metastatic CRC tissue. Eighteen CRC patients with lymph node and/or liver metastasis were selected, and hsa-miR-140-5p levels were quantified by real-time qRT-PCR and normalized to the internal control. The hsa-miR-140-5p levels in primary and metastatic tumor tissues were compared to corresponding normal tissues. **B.** Kaplan-Meier survival curves of 174 stage III and IV CRC patients were generated based on different expression level of hsa-miR-140-5p. HR = 0.49 (0.27–0.91); Cutoff value: median, *p* = 0.02)

Based on the above inhibitory effects of hsa-miR-140-5p on CRC and our previous study showing downregulation of hsa-miR-140-5p in CRC tissue as compared to normal colorectal mucosa [33], we further analyzed the expression level of hsa-miR-140-5p in metastatic CRC tissue. We selected 18 CRC patients with corresponding liver and/or lymph node metastasis, and extracted RNA from normal colorectal mucosa, tumor tissue and metastatic tumor tissue. After comparing hsa-miR-140-5p expression levels, we found that hsa-miR-140-5p is significantly downregulated in primary CRC tissue and further decreased in lymph node and liver metastasis tissue (Figure 6A). The expression level of hsa-miR-140-5p in normal tissue is around 2 fold higher than that in primary CRC tissue, and 5 fold higher than that in liver metastasis.

The prognostic potential of hsa-miR-140-5p was analyzed based on the miRNA expression data from 174 stage III or IV CRC patients in The Cancer Genome Atlas (TCGA). Kaplan-Meier survival analysis was performed based on the median value of hsa-miR-140-5p expression to divide the patients into two groups (high, low). Patients with high hsa-miR-140-5p expression had much longer survival time (131 months) than the low group (38 months) (Figure 6B). These results further support the notion that hsa-miR-140-5p may play important roles in CRC progression and metastasis, and it could be a potential therapeutic candidate and as a prognostic biomarker for metastatic CRC patients.

**Figure 7:**
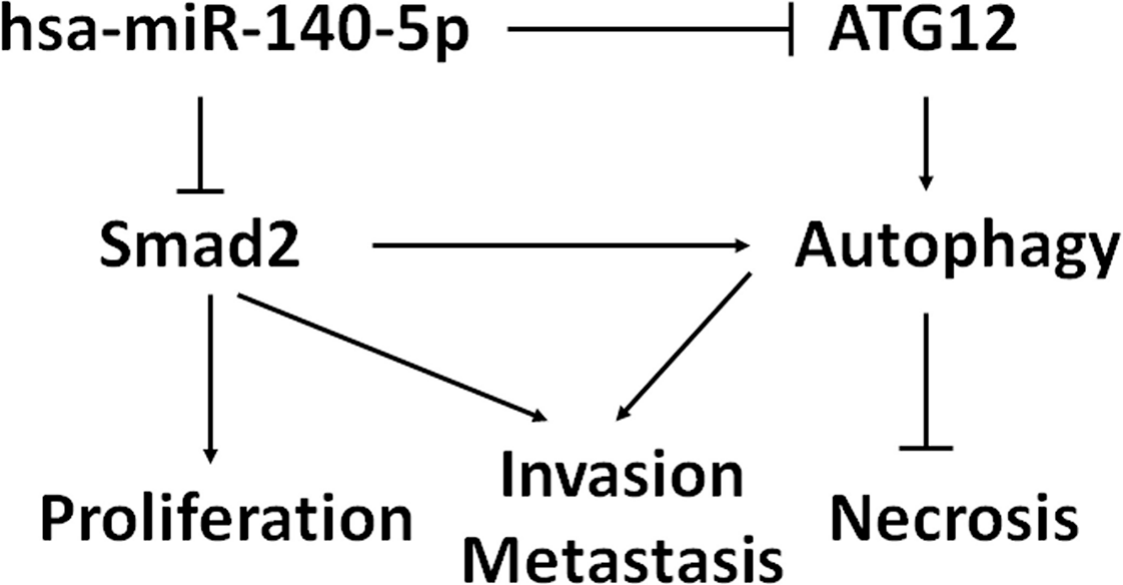
Proposed model of hsa-miR-140-5p function in CRC development and progression Hsa-miR-140-5p acts as a master regulator of colorectal cancer survival, invasion and metastasis through the suppression of Smad2. Hsa-miR-140-5p also inhibits colorectal CSC survival by suppressing ATG12 and disrupting autophagy.

Based on our results, a model was proposed for the potential roles of hsa-miR-140-5p in CRC (Figure 7). TGF-β regulates cell proliferation, invasion and autophagy in CRC through Smad2/Smad3 pathway. Hsa-miR-140-5p disrupts the TGF-β signaling pathway by directly targeting Smad2. Moreover, hsa-miR-140-5p down-regulates ATG12, a key regulator for autophagy. Additional targets mediated by hsa-miR-140-5p may also contribute to colon cancer growth and cell cycle. In summary, hsa-miR-140-5p elicits its inhibitory effects on CRC progression and metastasis through multiple targets and pathways.

## DISCUSSION

In this study, we discovered a novel epigenetic mechanism of Smad2 regulation by non-coding hsa-miR-140-5p in CRC through direct binding at the 3′-UTR of the Smad2 mRNA. Smad2 is one of the key downstream proteins in the TGF-β signaling pathway to regulate tumor invasion[8]. TGF-β regulates the growth, differentiation, and migration of various types of cells. It functions as both a tumor suppressor and a tumor promoter in cancer [8, 39]. In early stages of carcinogenesis, TGF-β serves as a tumor suppressor by inhibiting cell growth. However, in advanced cancer TGF-β facilitates the progression and metastasis of tumors through regulating epithelial-mesenchymal transition (EMT) [39]. It has been reported that hsa-miR-140-5p targets TGFBRI in HCC and suppresses HCC growth and metastasis [30]. However, TGFBRI is not the target of hsa-miR-140-5p in CRC based on our results. This suggests that the suppressing of TGFBRI by hsa-miR-140-5p may be disease specific. We show that Smad2 is the direct target of hsa-miR-140-5p and overexpression of hsa-miR-140-5p in CRC cell lines inhibited cell invasion (Figure 1). We further reveal that the inhibition of tumor invasion was directly due to the suppression of Smad2 by utilizing siRNA against Smad2 (Figure 2). As a consequence of inhibition of Smad2, we demonstrated that overexpression of hsa-miR-140-5p led to the reduction of invasion capacity in CRC cell lines. Consistent with our findings, hsa-miR-140-5p has been reported to participate in the regulatory network for cancer invasion in other cancer types [30, 32, 40].

The clinical relevance of hsa-miR-140-5p was clearly demonstrated by quantification of the expression levels of hsa-miR-140-5p in a set of 18 archival CRC patient samples of corresponding normal colorectal mucosa, primary tumor tissue and metastatic tumor tissue (Figure 6A). Consistent with our previous finding [33], we found hsa-miR-140-5p was progressively downregulated in CRC tumor tissues and further decreased in live and lymph node metastatic cancer tissues, suggesting that hsa-miR-140-5p is significantly correlated with CRC progression and metastasis (Figure 6A). It has been shown that Smad2 is upregulated in CRC cancer tissue as compared to normal colorectal mucosa [10, 11]. Such results are consistent with the reduction of hsa-miR-140-5p expression in CRC. The prognostic potential of hsa-miR-140-5p further supports the clinical significance in CRC (Figure 6B)

In addition to Smad2 and cancer invasion, our work revealed a novel regulatory function of hsa-miR-140-5p in CSC and autophagy. Autophagy is an essential and highly conserved critical catabolic process that delivers cytoplasmic components to lysosomes for degradation. There is mounting evidence to link autophagy and cancer [41, 42], and the role of autophagy in cancer progression is still unclear. Several reports demonstrated that activation of autophagy may suppress tumor development and lead to cell death [43, 44]. However it has been demonstrated extensively that autophagy mediates tumor survival by supplying nutrients to stressed cancer cells [45], and promotes cancer invasion through the TGF-β/Smad2/Smad3 signaling pathway [34, 35]. Furthermore, it has been shown that autophagy is an intrinsic metabolic feature of CSCs and it protects CSCs against microenvironment stress such as hypoxia and starvation, which is essential for CSCs maintenance [37]. Several miRNAs have been shown to regulate cancer progression through autophagy, such as miR-502, miR-30a [46–48]. Our research adds a new member to the miRNA family regulating autophagy. We demonstrated that hsa-miR-140-5p has two potential direct targets involved in the autophagic process, Smad2 and ATG12 (Figure 4C), and its overexpression had a great impact on CSC autophagic activity. Besides ATG12, an essential protein for autophagosome elongation [38], hsa-miR-140-5p also reduced the expression level of CTSB and CTSS (Figure 4C), which have been shown to participate in degradation of damaged organelles in autophagy [49]. In addition, CTSB is an important protease activating TGF-β [50], suggesting an indirect regulatory role of hsa-miR-140-5p on TGF-β signaling pathway. As a result of autophagy disruption, ectopic expression of hsa-miR-140-5p led to direct killing of CSCs *in vitro*.

Recently, mounting evidence suggests that CSCs are responsible for tumor initiation, metastases and chemoresistance [3]. More studies have been carried out to investigate potential miRNAs targeting CSCs as new anti-cancer therapeutic regimens. For example, in breast cancer, ectopic miR34a expression reduced cancer stem cell properties and increased sensitivity to doxorubicin treatment by directly targeting NOTCH1 [51]. In glioma stem-like cell lines HNGC-2 and NSG-K16, overexpression of miR-34a decreased the proliferative and migratory potential of these cells through Akt and Wnt signaling pathways [52]. We demonstrated that overexpression of hsa-miR-140-5p in colorectal CSCs reduced cell survival by inhibition of autophagy. The therapeutic potential of hsa-miR-140-5p was determined in mouse tumor models. Our results showed that ectopic expression of hsa-miR-140-5p in colorectal CSCs abolished the tumor initiation and metastasis capacity of these cells (Figure 5); suggesting hsa-miR-140-5p is a potential regimen as an adjuvant therapy for CRC patients. We realize that the lentiviral based delivery approach is not ideal for therapeutic development and it is just for demonstrating the proof-of-concept. In the future, we need to develop a more effective and optimized method to specifically target CRC cells for the delivering hsa-miR-140-5p *in vivo*.

In summary, we provided experimental evidence that hsa-miR-140-5p suppresses CRC progression and metastasis through the suppression of Smad2, a key player in the TGFβ signaling pathway (Figure 7). Hsa-miR-140-5p suppressed colon tumor initiation and metastasis *in vivo*. We identified a novel mechanism for hsa-miR-140-5p as a critical regulator of autophagy in colorectal CSCs. As a result, ectopic expression of hsa-miR-140-5p in CSCs disrupts the survival of colorectal CSCs. As miRNA based therapeutics are currently in clinical trials, our results suggest that hsa-miR-140-5p may offer a novel therapeutic strategy for treating CRC patients. Hsa-miR-140-5p also has a potential as a prognostic biomarker in advanced stages of CRC.

## MATERIALS AND METHODS

### Cell lines and reagents

The human CRC cell line HCT116 was kindly provided by Professor Bert Vogelstein (The Johns Hopkins University), and maintained in McCoy's 5A medium (Life Technologies). The other human colon cancer lines RKO and SW480 were purchased from the American Type Culture Collection (ATCC), and were maintained in DMEM medium (Life Technologies). All the media were supplemented with 10% fetal bovine serum (FBS, Sigma-Aldrich).

Colon CSCs were obtained by plating 10^6^ HCT116 cells in DMEM/F12 supplemented with B27, 10 ng/mL bFGF, and 20 ng/mL EGF (Life Technologies) in ultra-low attachment flasks for 2 weeks. The spheroid cells were collected by gentle centrifugation, dissociated to single cells, and cultured to generate next generation spheroids [53]. The spheroids were passed every week and CSC phenotype was confirmed with stem cell markers CD44 and CD133 by flow cytometry (Supplementary Figure 1A).

### miRNA and siRNA transfection

HCT116 cells, RKO, and SW480 cells were plated in 6-well plates at 2 × 10^5^ per well, respectively. For CSC transfection, single cell suspension was plated at 2 × 10^5^ cells/well in 6-well ultra-low attachment plates. Twenty-four hours after plating, 100 pmole of hsa-miR-140-5p precursor (Life Technologies) or siRNAs against Smad2 (Life Technologies) were transfected to the cells with oligofectamine (Life Technologies) following the manufacturer's protocol. Negative miRNA (Life Technologies) or scramble siRNA (Life Technologies) was also transfected as negative controls. The transfection efficiency was quantified by hsa-miR-140-5p qRT-PCR.

### Invasion assay

48 hours after transfection, cells were subjected to invasion assay using cell invasion assay kit (ECM550, Millipore) according to manufacturer's protocol. Briefly the inserts with an ECM layer were first rehydrated with serum-free DMEM media for 2 hours at room temperature. After rehydration, the media was carefully removed and 300 μl cell suspension containing 10^6^ cells/ml in serum-free media was added to the interior of the inserts. At the same time, 500 μl DMEM media with 10% FBS was added to the lower chamber. 24 hours later, the non-invading cells and ECM layer were removed by cotton-tipped swabs, and then the inserts were stained with 500 μl staining solution for 20 minutes. Then the inserts were rinsed with water and air dry. The invasion activity was quantified by dissolving stained cells with 10% acetic acid and colorimetric reading of OD at 560 nm. The experiments were repeated for 4 times.

### Clinical colorectal cancer samples

Eighteen colorectal cancer specimens were selected from patients with liver or lymph node metastasis, who underwent surgical resection of primary tumors at the Stony Brook University Medical Center, Stony Brook, NY, USA. Patient consent forms were obtained from each patient according to institutional policies. Patient clinical information was provided by the Cancer Registry of Stony Brook University Medical Center, and the characteristics of these patients are shown in Supplementary Table 1. Each patient sample contains normal colon mucosa, primary tumor, liver metastasis or lymph node metastasis. Representative tissue blocks from each case were assembled from the archival collections of the Department of Pathology, and used for subsequent analysis.

From the archival FFPE tissues, areas of primary or metastasizing colon cancer were identified using the corresponding hematoxylin and eosin (H&E) stained sections and cores measuring 1.5 mm in diameter and 2 mm in length (approximately 0.005 g) were extracted. Then the samples were deparaffinized with xylenes, hydrated by using decreasing concentrations of ethanol, and digested with proteinase K. Total RNA was isolated with Trizol reagent according to the manufacture's protocol (Life Technologies).

We performed overall survival analyses, based on 174 stage III or IV CRC patients from the TCGA project. The clinical and miRNA expression data were downloaded from the UCSC cancer genome browser (https://genome-cancer.ucsc.edu) [54]. The expression profile of the hsa-miR-140-5p among the 174 patients was extracted, based on which we divided the patients into two groups (high, low): expression greater than the median expression of the patients was denoted as “high;” otherwise it was denoted “low.” Subsequently, we performed log-rank tests between the two groups and generated corresponding Kaplan-Meier curves.

### Luciferase assay

A 2 Kb fragment of the 3′UTR of Smad2 containing predicted binding sites for hsa-miR-140-5p was cloned from human genomic DNA and inserted into pMIR-REPORT plasmid (Life Technologies). Twenty-four hours before transfection, 1.5 × 10^4^ cells were plated in a 96-well plate. 10 pmole of hsa-miR-140-5p or negative miRNA was transfected into cells together with 100ng of pMIR-REPORT-3UTRSmad2 and 1ng of Renilla luciferase plasmid pRL-SV40 (Promega) by DharmaFect Duo (Dharmacon). Luciferase assay was performed 24 hours after transfection by the dual-luciferase reporter assay system (Promega). For each sample, firefly luciferase activity was normalized to Renilla luciferase activity and the inhibition of hsa-miR-140-5p on Smad2 3′-UTR was normalized to the control miRNA.

### Lentivirus production

All the materials for lentivirus production were purchased from GeneCopoeia. Briefly, 1.5 × 10^6^ 293T cells were plated in 10-cm dish with 10ml of DMEM+10% FBS. Two days later, pEZX-MR03, a lentiviral plasmid, expressing hsa-miR-140-5p was transfected with Lenti-Pac HIV expression packaging kit following the manufacturer's protocol. 48 hours later, virus was harvested and concentrated with Lenti-Pac lentivirus concentration solution. Then the titer of the virus (approximately 10^11^ virus particles/ml) was determined with Lenti-Pac HIV qRT-PCR titration kit. In addition, serial dilution of the virus (0.1 μl, 0.5 μl, 2 μl, 10 μl, 50 μl) was used to transduce 5 × 10^4^ HCT116 CSC to determine the transduction efficiency. The lowest concentration (2 μl) to achieve 100% positive expression was used to infect the cells for mouse *in vivo* experiments.

### Mouse subcutaneous tumor implantation and colon cancer metastasis model

Two days before injection, HCT116 CSCs were plated at 5 × 10^5^/well in 6-well ultra low attachment plate. 20 μl of the virus or 100 pmole miRNA precursors was used to transduce or transfect cells. 48 hours later, cells were collected and resuspended at 10^6^/ml in DMEM/F12 knockout media with 30% matrigel. Ten-twelve week old NOD/SCID mice (Jackson Laboratories, Bar Harbor, MA, USA) were used for tumor implantation. All animal procedures were approved by the Stony Brook University Institutional Animal Care and Use Committee (IACUC). The mice were anesthetized by isoflurane inhalation. 100 μl of cell suspension was injected subcutaneously into both sides of lower back area. The tumor size was measured using a caliper and tumor volume was calculated using the formula V = length x width^2^/2 [47, 55]. For mouse colon cancer metastasis model, cells were collected and resuspended at 10^6^/ml in DMEM/F12 knockout media and 100 μl was injected through tail vein [56]. All the mice were euthanized on 8 weeks after injection by CO_2_ inhalation. And the subcutaneous tumors, livers and lungs were collected for further analysis.

### Statistical analysis

All experiments were repeated at least three times. All statistical analyses were performed with Graphpad Prism software. The statistical significance between two groups was determined using Student's *t*-test (paired *t*-test for clinical samples, and unpaired *t*-test for all other samples). For comparison of more than two groups, one-way ANOVA followed by a Bonferroni-Dunn test was used. Data were expressed as mean ± standard error of the mean (SEM). The statistical significance is either described in figure legends, or indicated with asterisks (*). * = *P* < 0.05; ** = *P* < 0.01; *** = *P* < 0.001.

## SUPPLEMENTARY MATERIAL METHODS


