# Supplementary material for: Inhibition of colorectal cancer stem cell survival and invasive potential by hsa-miR-140-5p mediated suppression of Smad2 and autophagy
Source: Oncotarget. 2015 Apr 27;6(23):19735–46. doi: 10.18632/oncotarget.3771 (PMC4637317; doi:10.18632/oncotarget.3771)
Supplement: Supplementary file 1 [file oncotarget-06-19735-s001.pdf]

SUPPLEMENTARY MATERIALS AND METHODS

Cell proliferation assay

Twenty-four hours after transfection, HCT116, RKO and SW480 cells were seeded in 96-well plates at 2,000 or 4,000 cells per well, respectively. The cell proliferation assay was performed on days 1, 3 and 5 by incubating 10 µl WST-1 (Roche Applied Science) in the culture medium for one hour and reading the absorbance at 450 and 630 nm [1, 2]. The cell proliferation rate was calculated by subtracting the absorbance at 450 nm from the obsorbance at 630 nm.

Cell cycle analysis

Thirty-six hours after transfection, cells were harvested and resuspended at 0.5 to 1 × 10^6 cells/ml in modified Krishan buffer with 0.02 mg/ml RNase H (Life Technologies) and 0.05 mg/ml propidium iodide (PI, Sigma-Aldrich) [3, 4]. The samples were analyzed by flow cytometry and results were calculated with Modfit LT™ software.

RNA extraction and real-time quantitative RT-PCR (qRT-PCR) analysis of mRNA and miRNA

Total RNA, including miRNAs, was isolated from clinical specimens, and cell lines 24 hours after transfection or treatment by using the TRIzol reagent (Life Technologies) following manufacturer protocol. All reagents for real-time qRT-PCR were ordered from Applied Biosystems. 1µg of RNA was used as a template for cDNA synthesis with high capacity cDNA synthesis kit and random primers. Then the cDNA templates were mixed with gene-specific primers for Smad2 and internal control GAPDH and Taqman 2x universal PCR master mix. Applied Biosystems 7500 Real-Time PCR machine was used for q-RT-PCR and programmed as: 50°C, 2 minutes; 95°C, 10 minutes; 95°C, 15 seconds; 60°C, 1 minute and the latter three steps were repeated for 40 cycles. For quantification of hsa-miR-140-5p, 10ng of RNA was used as a template and cDNA was synthesized with miRNA-specific primers. Similarly to mRNA qRT-PCR, the hsa-miR-140-5p level was analyzed with its specific primers and internal control. Fluorescent signals from each sample were collected at the endpoint of every cycle, and the expression level of genes and hsa-miR-140-5p was calculated by ΔC_T values based on the internal controls, normalized to the control group and plotted as relative value (RQ).

Microarray and cell death pathway finder PCR array

The RNA samples, extracted from HCT116 cells transfected with hsa-miR-140-5p or negative miRNA, were sent to Ocean Ridge Biosciences, FL, USA for microarray analysis.

The RNA samples, extracted from HCT116 CSCs cells transfected with hsa-miR-140-5p or negative miRNA, were transcribed to first-strand cDNA using the RT^2 First Strand Kit (SABiosciences, Qiagen). Next, the cDNA was mixed with RT^2 SYBR Green Mastermix (SABiosciences), and this mixture was aliquoted into the wells of the Cell Death PathwayFinder PCR Array (PAHS-212Z). Applied Biosytems 7500 Real-Time PCR machine was used for qRT-PCR, and relative expression values were determined using the ΔΔCT method.

Western blot

The protein samples were collected 48 hours after transfection with RIPA buffer (Sigma-Aldrich). Equal amounts of samples were loaded to SDS-PAGE, then transferred to polyvinylidene difluoride (PVDF) membrane. The membranes were blocked with 5% non-fat milk in TBS/0.5% Tween-20 (TBS-T) at room temperature for one hour, then probed with mouse anti-human Smad2 antibody (1:1000, Cell Signaling), and mouse anti-human GAPDH antibody (1:1000, Santa Cruz overnight at 4°C. Goat anti-rabbit and anti-mouse horseradish peroxidase (HRP)-conjugated secondary antibodies (Jackson Immunoresearch) were used at 1:5000. HRP activity was detected with SuperSignal West Pico Chemiluminescent Substrate (Thermo Scientific) and visualized in an UVP Bioimaging system. Expression levels were quantified using ImageJ software, and normalized to loading controls.

REFERENCES

1. Song B, et al. Molecular mechanism of chemoresistance by miR-215 in osteosarcoma and colon cancer cells. Mol Cancer. 2010; 9:96.
2. Song B, et al. Mechanism of chemoresistance mediated by miR-140 in human osteosarcoma and colon cancer cells. Oncogene. 2009; 28:4065–74.
3. Krishan A. Rapid flow cytofluorometric analysis of mammalian cell cycle by propidium iodide staining. J Cell Biol. 1975; 66:188–93.
4. Dressler L.G. et al. DNA flow cytometry and prognostic factors in 1331 frozen breast cancer specimens. Cancer. 1988; 61:420–7.

SUPPLEMENTARY FIGURES AND TABLES

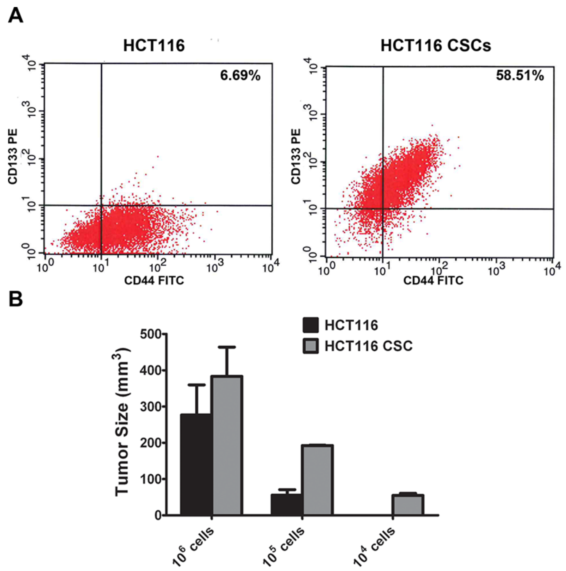

Supplementary Figure S1: Characteristics of CSCs isolated from HCT116 cells. A. Representative images of flow cytometry analysis with CD44 and CD133 antibody. **B.** CSCs can form subcutaneous tumors in NOD/SCID mice at much lower cell numbers when compared to parental HCT116 cells. Final tumor sizes were measured at 4 weeks after injection.

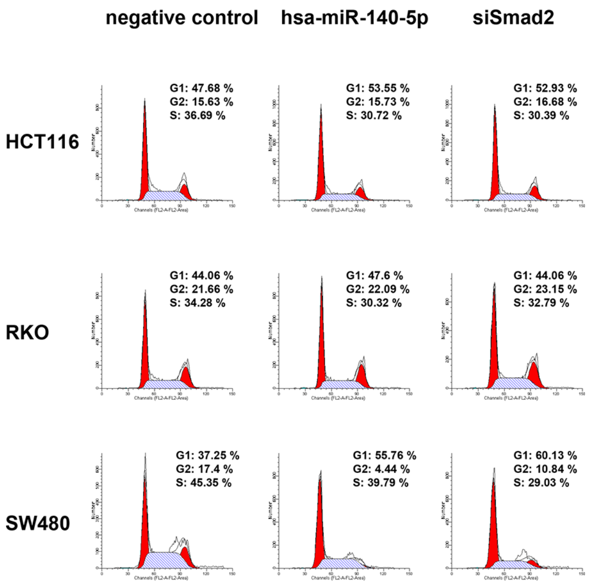

Supplementary Figure S2: Overexpression of hsa-miR-140-5p in CRC cells induced cell cycle arrest. Representative images of Modfit LT™ software analysis are shown.

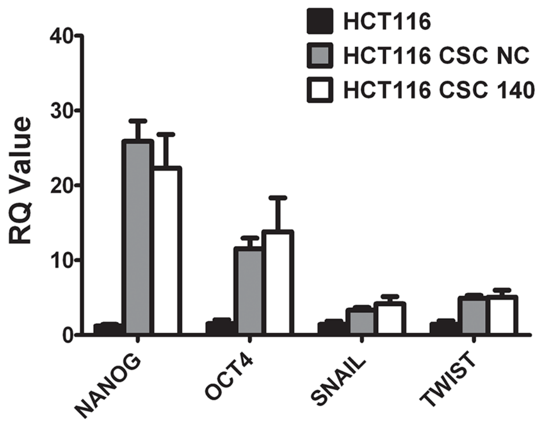

Supplementary Figure S3: Overexpression of hsa-miR-140-5p in CSCs did not impact stemness markers. 48 hours after transfection of CSCs with hsa-miR-140-5p precursors, total RNA was extracted, and the expression levels of NANOG, OCT4, Snail and Twist were quantified with qRT-PCR.

Supplementary Table S1: Clinicopathological features of 18 CRC patients

Characteristics		Frequency	Percentage (%)
**Age**	Median	58.5 (45 – 77)	
**Gender**	Female	6	33.3
	Male	12	66.7
**Tumor localization**	Colon	17	94.4
	Rectum	1	5.56
**TNM**	3	12	66.7
	4	6	33.3
**pT**	1	1	5.56
	2	0	0
	3	12	66.7
	4	5	27.8
**pN**	0	1	5.56
	1	8	44.4
	2	9	50
**Metastasis**	No	12	66.7
	Yes	6	33.3

Supplementary Table S2: Direct targets of hsa-miR-140-5p validated by microarray and Targetscan

Gene_Symbol	Fold Changes (hsa-miR-140-5p vs. negative control)
RAB17	0.01
FRAS1	0.02
NUDT11	0.02
SLMAP	0.03
SPOCK1	0.03
DZIP1	0.04
SLC6A6	0.11
KIF1B	0.13
CRHR1	0.17
NTN4	0.17
GNA13	0.19
BCL2	0.22
TLR4	0.23
CD99L2	0.24
TGM2	0.26
PANK1	0.31
MMD	0.33
KCNH1	0.33
PROM2	0.34
EMR2,CD97	0.34
MASP1	0.35
GPR161	0.35
STOM	0.37
TSPAN12	0.37
SBF1	0.40
ATG12	0.41
CDC42EP3	0.41
SLC22A17	0.41
ITPKB	0.42
KLF6	0.43
ARHGAP19	0.43
RAB31	0.45
ARHGAP31	0.46

(*Continued*)

Gene_Symbol	Fold Changes (hsa-miR-140-5p vs. negative control)
RAB10	0.46
PTP4A3	0.47
PDE2A	0.47
RBM39	0.48
PARD6B	0.48
PFKFB4	0.48
SLC16A14	0.49
